# Assessment of mortality-related risk factors and effective antimicrobial regimens for treatment of bloodstream infections caused by carbapenem-resistant *Pseudomonas aeruginosa* in patients with hematological diseases

**DOI:** 10.3389/fcimb.2023.1156651

**Published:** 2023-06-21

**Authors:** Sisi Zhen, Yuanqi Zhao, Zhangjie Chen, Tingting Zhang, Jieru Wang, Erlie Jiang, Fengkui Zhang, Yingchang Mi, Xiaofan Zhu, Mingzhe Han, Zhijian Xiao, Jianxiang Wang, Sizhou Feng

**Affiliations:** ^1^ State Key Laboratory of Experimental Hematology, National Clinical Research Center for Blood Diseases, Haihe Laboratory of Cell Ecosystem, Institute of Hematology & Blood Diseases Hospital, Chinese Academy of Medical Sciences & Peking Union Medical College, Tianjin, China; ^2^ Tianjin Institutes of Health Science, Tianjin, China; ^3^ Fujian Institute of Hematology, Fujian Provincial Key Laboratory on Hematology, Fujian Medical University Union Hospital, Fuzhou, China

**Keywords:** multidrug-resistant *Pseudomonas aeruginosa*, bacteremia, ceftazidime-avibactam, hematological diseases, carbapenem-resistant *Pseudomonas aeruginosa*

## Abstract

**Background:**

Infections caused by carbapenem-resistant *Pseudomonas aeruginosa* (CRPA) are related to higher mortality. The objective of this study was to explore clinical outcomes of CRPA bacteremia, identify risk factors and also, compare the efficacy of traditional and novel antibiotic regimens.

**Methods:**

This retrospective study was conducted at a blood diseases hospital in China. The study included hematological patients who were diagnosed with CRPA bacteremia between January 2014 and August 2022. The primary endpoint was all-cause mortality at day 30. Secondary endpoints included 7-day and 30-day clinical cure. Multivariable Cox regression analysis was employed to identify mortality-related risk factors.

**Results:**

100 patients infected with CRPA bacteremia were included and 29 patients accepted allogenic-hematopoietic stem cell transplantation. 24 received ceftazidime-avibactam (CAZ-AVI)-based therapy and 76 received other traditional antibiotics. 30-day mortality was 21.0%. Multivariable cox regression analysis showed neutropenia >7 days after bloodstream infections (BSI) (P=0.030, HR: 4.068, 95%CI: 1.146~14.434), higher Pitt bacteremia score (P<0.001, HR:1.824, 95%CI: 1.322~2.517), higher Charlson comorbidity index (P=0.01, HR: 1.613, 95%CI: 1.124~2.315) and bacteremia due to multidrug-resistant *Pseudomonas aeruginosa* (MDR-PA) (P=0.024, HR:3.086, 95%CI: 1.163~8.197) were identified as independent risk factors of 30-day mortality. After controlling for confounders, an additional multivariable cox regression analysis revealed definitive regimens containing CAZ-AVI were associated with lower mortality in CRPA bacteremia (P=0.016, HR: 0.150, 95%CI: 0.032~0.702), as well as in MDR-PA bacteremia (P=0.019, HR: 0.119, 95%CI: 0.020~0.709).

**Conclusions:**

For patients with hematological diseases and CRPA bacteremia, 30-day mortality rate was 21.0% (21/100). Neutropenia >7 days after BSI, higher Pitt bacteremia score, higher Charlson comorbidity index and bacteremia due to MDR-PA increased 30-day mortality. CAZ-AVI-based regimens were effective alternatives for bacteremia due to CRPA or MDR-PA.

## 1 Introduction

*Pseudomonas aeruginosa* is an important nosocomial pathogen responsible for a diverse range of infections, including pneumonia, bacteremia and urinary tract infections, especially for seriously ill patients (Lister et al., 2009). Carbapenem was previously regarded as the last resort for these infections. Nonetheless, the widespread utilization of carbapenem antibiotics caused a more severe complication, namely the emergence of carbapenem-resistant *Pseudomonas aeruginosa* (CRPA) and multidrug resistant *Pseudomonas aeruginosa* (MDR-PA). A large number of studies have investigated the relationship between carbapenem resistance and mortality and found that comparing to susceptible *Pseudomonas aeruginosa*, infections due to CRPA or MDR-PA were associated with higher mortality, as well as hospital costs (Tam et al., 2010; Zhang et al., 2016; Chen et al., 2019; Shi et al., 2019). Our prior study also demonstrated that among patients with acute leukemia, individuals who developed bloodstream infections (BSI) caused by MDR-PA experienced a significantly higher 30-day mortality rate comparing to those with non-MDR-PA BSI (28.9% vs 5.5%, P<0.001) (Zhao et al., 2020). WHO has identified CRPA as a critical priority pathogen due to its rising prevalence, higher mortality rates and prolonged hospitalization.

Patients with hematological diseases are vulnerable to antibiotic-resistant *Pseudomonas aeruginosa* infections. This vulnerability is attributed to distinctive disease characteristics, long stay of hospital, neutropenia, impairment of immune system and long-term use of antibiotics. Understanding the clinical characteristics, antibiotic resistance, risk factors of outcomes and choosing effective therapy is crucial for improving the survival of these infections. However, information about CRPA and MDR-PA BSI exclusively for patients with hematological diseases are limited. This retrospective study was carried out to report clinical outcomes of CRPA BSI, identify risk factors of mortality and also, compare the impact of different antimicrobial regimens.

## 2 Patients and methods

### 2.1 Setting and study design

**Figure 1:**
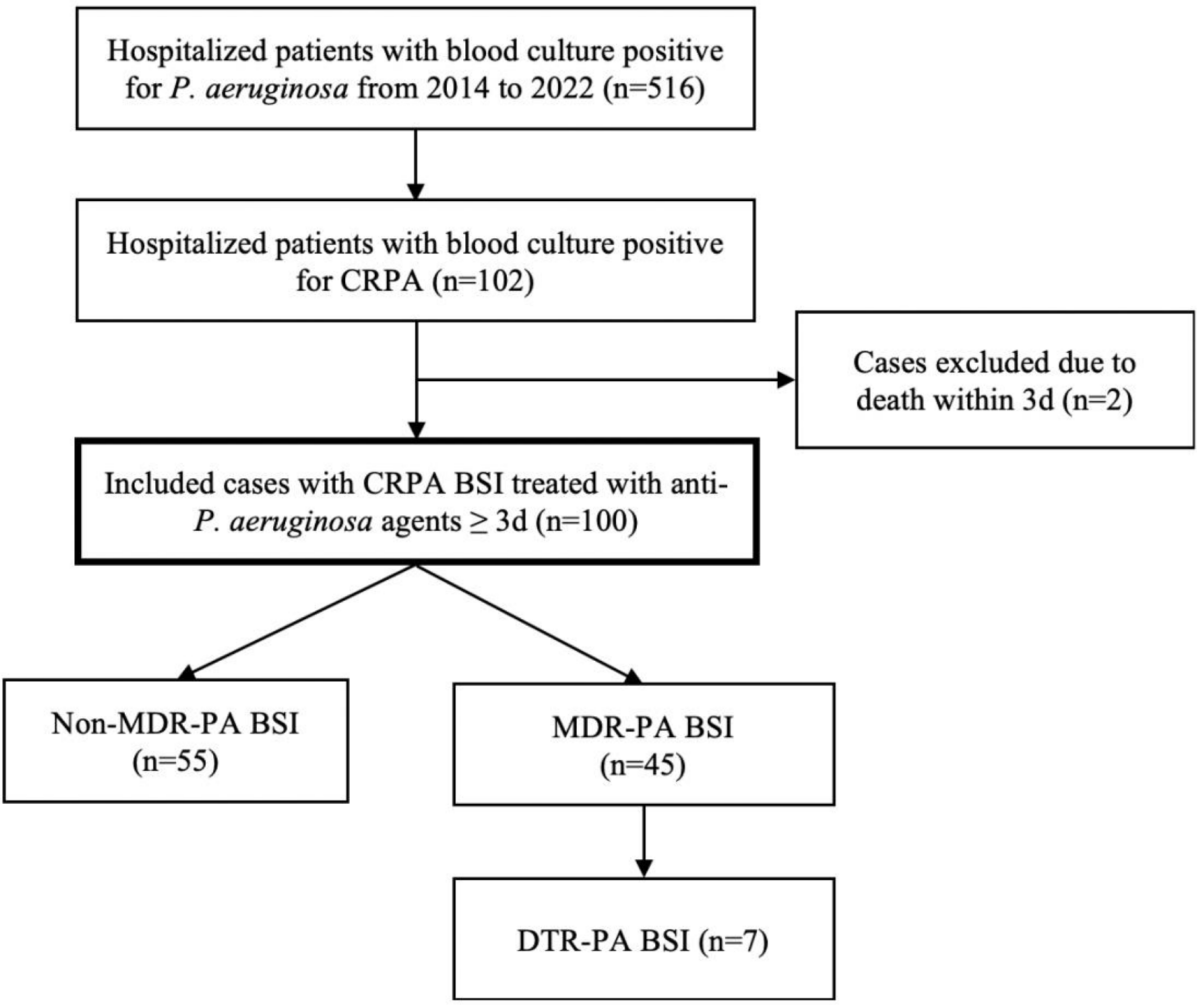
Study Flow Chart. CRPA, carbapenem-resistant Pseudomonas aeruginosa; BSI, bloodstream infections; MDR-PA, multidrug-resistant Pseudomonas aeruginosa; DTR-PA, difficult to treat Pseudomonas aeruginosa.

An observational cohort study was carried out retrospectively at a blood diseases hospital in Tianjin, China. This study included hematological patients who were definitely diagnosed as CRPA BSI (the positive culture from blood sample) and treated with anti-*Pseudomonas aeruginosa* agents at least 72 h between January 2014 and August 2022. Patients who died within 72 h were excluded (**Figure 1**). All data in our study were obtained from the electronic medical record system. The primary endpoint was all-cause mortality at day 30. Secondary endpoints included 7-day and 30-day clinical cure. The present study obtained approval from the ethical committee of the Institute of Hematology and Blood Diseases Hospital, Chinese Academy of Medical Sciences.

### 2.2 Variables and definitions

The data obtained included demographics, underlying diseases, comorbidities, microbiologic data, infection-related characteristics, antibiotic treatment characteristics and clinical outcomes. CRPA was defined as an isolate with Minimum Inhibitory Concentration (MIC) ≥ 4 ug/mL for imipenem or meropenem according to 2021 Clinical and Laboratory Standards Institute (CLSI) guidelines (CLSI, 2021). MDR-PA was defined as nonsusceptibility to at least three of the following antimicrobial categories: aminoglycosides, antipseudomonal carbapenems, antipseudomonal cephalosporins, antipseudomonal fluoroquinolones, antipseudomonal penicillins with β-lactamase inhibitors or polymyxins (Magiorakos et al., 2012). Additionally, difficult to treat (DTR)-PA was defined as nonsusceptibility to all of the following: ceftazidime, cefepime, piperacillin-tazobactam, aztreonam, imipenem-cilastatin, meropenem, ciprofloxacin and levofloxacin according to the guidance (Kadri et al., 2018). Neutropenia > 7 days meant the count of neutrophil was less than 0.5×10^9^/L persistently for more than 7 days from onset of CRPA BSI. The Charlson comorbidity index was determined on admission. The Pitt bacteremia score was calculated at BSI onset. Empirical therapy was described as administration of antibiotics prior to availability of *in vitro* antibiotic susceptibility testing to the clinician. On the other hand, definitive therapy referred to administration of antibiotics once these results had been reported. Finally, combination therapy was characterized as receiving at least two *Pseudomonas aeruginosa* targeted antibiotics for 72 hours or more. Clinical cure referred to survival, disappearance of clinical signs and symptom such as fever, and without relapse of infections. Relapse was recurrent of bacteremia with the same species and similar susceptibility pattern after at least one negative growth of microorganisms.

### 2.3 Antimicrobial susceptibility and carbapenemase detection

An automated system (VITEK 2 Compact) was used for strain identification and routine drug sensitivity test. According to 2021 guidelines of CLSI M100, the antibiotic susceptibilities were defined (CLSI, 2021). *Pseudomonas aeruginosa* ATCC27853 was standard quality control strains, which were purchased from the National Health and Wellness Commission Clinical Inspection Center. This study employed disk diffusion method to determine the susceptibility of bacteria to ceftazidime-avibactam (CAZ-AVI).

CRPA strains from clinical samples between October 2017 and April 2021 were performed carbapenemase detection. Carbapenemase gene (including KPC, IMP, NDM, VIM, OXA-48, OXA-23) was tested by polymerase chain reaction (PCR) (Poirel et al., 2011). PCR instrument (VeritiTM DX96 Well Thermal Cycler) was purchased from Shanghai, China (Thermo Fisher Scientific Co., Ltd). PCR amplification products were subjected to agarose gel electrophoresis using electrophoresis apparatus–JY300C (purchased from Beijing JUNYI Electrophoresis Co., Ltd), detected using JQHX-5000 (purchased from Shanghai Jingqi Instrument Co., Ltd).

### 2.4 Statistical analysis

Continuous variables were reported as median and interquartile range (IQR), while categorical variables were presented as absolute numbers and corresponding percentages. Mann-Whitney U-test was employed to evaluate the relationship between continuous variables that were not normally distributed. In contrast, chi-square test was used to assess the association between categorical variables (using Fisher’s exact test instead of chi square when there were < 5 patients per cell). Variables with P values less than 0.05 in univariable analysis were involved in subsequent multivariable analysis. Multivariable cox regression was conducted to assess risk factors for mortality. The survival curves were generated by Kaplan-Meier survival estimates. Multivariable cox regression was performed by backward stepwise selection. The statistical tests in the analysis were two-tailed, with a significance level set at P value < 0.05. Statistical analyses were performed by SPSS version 26.0 (IBM, Corp).

## 3 Results

### 3.1 Baseline characteristics

100 patients with hematological diseases infected with CRPA BSI were included. The most common primary disease was acute myeloid leukemia (n=51), followed by acute lymphocytic leukemia (n=28), aplastic anemia (n=8) and others. The median age was 38.5 (IOR: 26.3~51.8) years, and 56.0% (n=56) were male. The median of Pitt bacteremia score was 1.0 (IQR: 0~1.8, range: 0~6.0) and median of Charlson comorbidity index was 2.0 (IQR: 2.0~3.0, range: 1.0~6.0). The median counts of leucocyte were 0.15 (IQR: 0.53~0.59) ×10^9^/L at the day of positive blood culture. Central venous catheters (CVC) were placed in 91 patients but only 2 case was considered as CVC-associated bacteremia.

A total of 29 patients accepted allogenic hematopoietic stem cell transplantation, with the donors being human leukocyte antigen (HLA) -matched related individuals (n=11), HLA-matched unrelated individual (n=1) and haploidentical individuals (n=17). 17 were diagnosed with CRPA BSI during the period of bone marrow suppression either before or after transplantation. The median duration from occurrence of transplantation to blood culture positivity was observed to be 6 days (IQR: -1~9, range: -6~11).

### 3.2 Antimicrobial susceptibility and carbapenemase detection results

**Table 1**; **Figure 2** showed resistance profiles of the 20 antimicrobial agents used for treatment of infections due to *Pseudomonas aeruginosa*. The antimicrobial nonsusceptibility to meropenem, imipenem and doripenem were 95.96% (95/99), 95.00% (95/100) and 77.66% (74/94). More than 70% CRPA were susceptible to colistin, tobramycin, amikacin, gentamycin, CAZ-AVI, ciprofloxacin, cefepime, ceftazidime and levofloxacin. Of these 100 CRPA strains, 45 (45.0%) were MDR-PA and 7 (7.0%) were DTR-PA. For MDR-PA strains, the rankings of the top five susceptible rates were colistin (100.00%), tobramycin (93.33%), amikacin (93.33%), gentamycin (88.24%) and CAZ-AVI (76.92%) (**Table 2**).

**Table 1:** Susceptibility of CRPA (n=100) to Antimicrobial Agents.

Antimicrobial Agents	Tested N	Susceptibility N (%)	Intermediate N (%)	Resistance N (%)
Meropenem	99	4 (4.04)	36 (36.36)	59 (59.60)
Imipenem	100	5 (5.00)	1 (1.00)	94 (94.00)
Doripenem	94	21 (22.34)	20 (21.28)	53 (56.38)
Gentamicin	77	73 (94.81)	2 (2.60)	2 (2.60)
Tobramycin	100	97 (97.00)	0 (0.00)	3 (3.00)
Amikacin	100	97 (97.00)	1 (1.00)	2 (2.00)
Colistin	23	23 (100.00)	0 (0.00)	0 (0.00)
Doxycycline	52	1 (1.92)	0 (0.00)	51 (98.08)
Ciprofloxacin	100	82 (82.00)	15 (15.00)	3 (3.00)
Levofloxacin	100	71 (71.00)	10 (10.00)	19 (19.00)
Ceftazidime	99	71 (71.72)	11 (11.11)	17 (17.17)
Cefepime	98	71 (72.45)	8 (8.16)	19 (19.39)
Piperacillin-tazobactam	87	57 (65.52)	17 (19.54)	13 (14.94)
Cefoperazone-sulbactam	89	51 (57.30)	26 (29.21)	12 (13.48)
Ticarcillin clavulanic acid	93	29 (31.18)	24 (25.81)	40 (43.01)
Ceftazidime-Avibactam	29	25 (86.21)	0 (0.00)	4 (13.79)

CRPA, carbapenem-resistant Pseudomonas aeruginosa.

**Figure 2:**
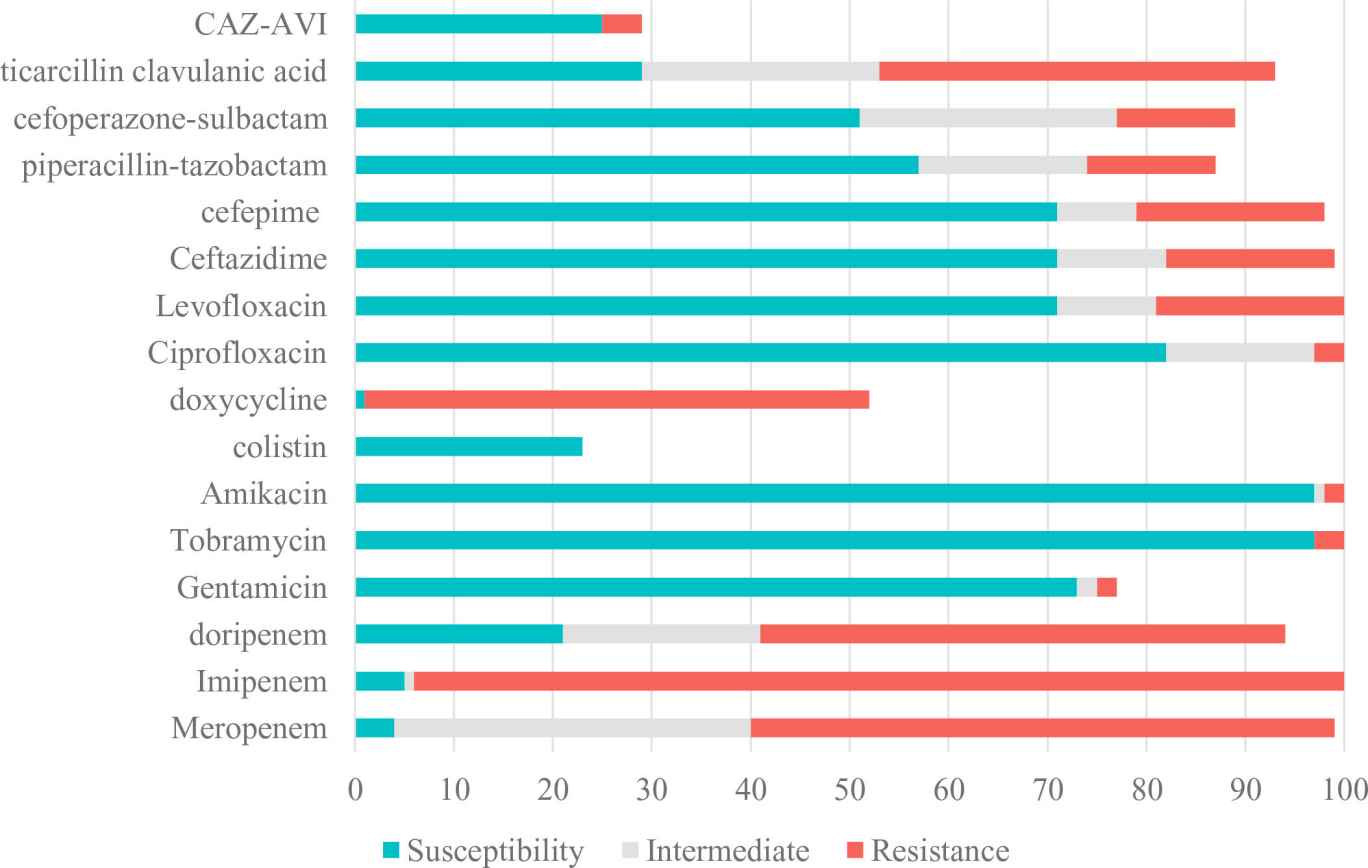
Susceptibility of CRPA to Antimicrobial Agents. CRPA, carbapenem-resistant Pseudomonas aeruginosa; CAZ-AVI, ceftazidime-avibactam.

**Table 2:** Susceptibility of MDR-PA (n=45) to Antimicrobial Agents.

Antimicrobial Agents	Tested N	Susceptibility N (%)	Intermediate N (%)	Resistance N (%)
Meropenem	44	1 (2.27)	6 (13.64)	37 (84.09)
Imipenem	45	4 (8.89)	1 (2.22)	40 (88.89)
Doripenem	44	5 (11.36)	4 (9.09)	35 (79.55)
Gentamicin	34	30 (88.24)	2 (5.88)	2 (5.88)
Tobramycin	45	42 (93.33)	0 (0.00)	3 (6.67)
Amikacin	45	42 (93.33)	1 (2.22)	2 (4.44)
Colistin	11	11 (100.0)	0 (0.00)	0 (0.00)
Doxycycline	31	0 (0.00)	0 (0.00)	31 (100.00)
Ciprofloxacin	45	29 (64.44)	13 (28.89)	3 (6.67)
Levofloxacin	45	18 (40.00)	10 (22.22)	17 (37.78)
Ceftazidime	45	17 (37.78)	11 (24.44)	17 (37.78)
Cefepime	44	18 (37.78%)	7 (15.91)	19 (43.18)
Piperacillin-tazobactam	35	11 (31.43)	12 (34.29)	12 (34.29)
Cefoperazone-sulbactam	39	16 (41.03)	12 (30.77)	11 (28.21)
Ticarcillin clavulanic acid	42	1 (2.38)	6 (14.29)	35 (83.33)
Ceftazidime-Avibactam	13	10 (76.92)	0 (0.00)	3 (23.0)

MDR-PA, multidrug-resistant Pseudomonas aeruginosa.

A total of 274 CRPA from clinical samples were collected to identify carbapenemase gene between October 2017 and April 2021. The majority isolates were obtained from throat swabs (23.7%, 65/274), followed by anal swabs (23.7%, 65/274) and blood (20.8%, 57/274). Only 4.7% (13/274) were found to produce carbapenemase, with IMP being the most common carbapenemase detected in 12 isolates, followed by NDM in one isolate. It is noteworthy that none of the bloodstream-derived strains were found to express carbapenemase.

### 3.3 Antibiotic regimens

83 patients received empirical monotherapy, including carbapenem (n=65), traditional β-lactam/β-lactamase inhibitor (n=11), cephalosporins (n=3), aminoglycosides (n=2), tigecycline (n=1) and CAZ-AVI (n=1), whereas others received empirical combination therapy. Clinicians usually adjusted antibiotic regimens according to *in vitro* antibiotic susceptibility testing. 57.0% (57/100) patients received *in vitro* active antibiotics within 48 h from onset of BSI. The median duration between BSI onset and initiation of *in vitro* active treatment was 2 days (IQR: 1~3, range: 1~10). The median time of active antimicrobial therapy duration was 10 days (IQR: 6.25~13, range: 3~33). 24 patients received treatment with CAZ-AVI-based regimens, while 76 with others. 41 individuals received definitive monotherapy. The different definitive antimicrobial regimens were shown in **Table 3**.

**Table 3:** The outcomes of different definitive antimicrobial regimen in patients with CRPA BSI (n=100).

Treatment regimen	Mortality (%)
Monotherapy	
CAZ-AVI	1/6 (16.67%)
Quinolones	0/1 (0)
Carbapenems	1/18 (5.56%)
Traditional β-lactam/β-lactamase inhibitor	3/13 (23.08%)
Colistin	1/2 (50.00%)
Cephalosporin	0/1 (0)
Double drugs combination	
CAZ-AVI + Carbapenems	1/2 (50.00%)
CAZ-AVI + Aminoglycosides	0/1/ (0)
CAZ-AVI + Quinolones	0/5 (0)
CAZ-AVI + Aztreonam	0/7 (0)
Traditional β-lactam/β-lactamase inhibitor + Carbapenems	0/2 (0)
Traditional β-lactam/β-lactamase inhibitor + Aminoglycosides	0/4 (0)
Traditional β-lactam/β-lactamase inhibitor + Quinolones	3/15 (20.00%)
Quinolones + Aminoglycosides	3/4 (75.00%)
Quinolones + Cephalosporin	1/2 (50.00%)
Carbapenems + Aminoglycosides	1/2 (50.00%)
Colistin + Quinolones	1/2 (50.00%)
Colistin + Cephalosporin	1/1 (100.00%)
Colistin + Aztreonam	1/1 (100.00%)
Cephalosporin + Aminoglycosides	0/2 (0)
Triple drugs combination	
CAZ-AVI-based regimens	0/3 (0)
Other regimens	3/6 (50.00%)

CAZ-AVI, ceftazidime-avibactam.

### 3.4 Clinical outcomes and risk factors for mortality

#### 3.4.1 For CRPA

**Figure 3:**
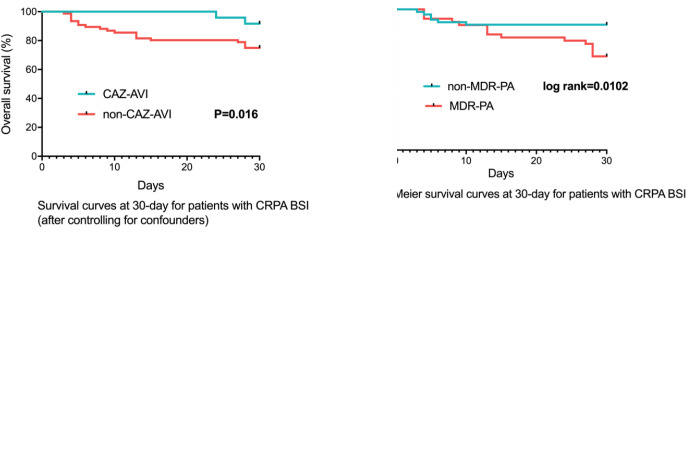
Survival Rates of CRPA BSI Patients (n=100). CRPA, carbapenem-resistant Pseudomonas aeruginosa; BSI, bloodstream infections; MDR-PA, multidrug-resistant Pseudomonas aeruginosa; CAZ-AVI, ceftazidime-avibactam.

For 100 CRPA bacteremia infection patients, all-cause mortality at day 30 was 21.0% (21/100). Clinical cure at 7 days and 30 days were achieved in 63.0% (63/100) and 77.0% (77/100) of patients, respectively. The different definitive antimicrobial regimens and outcomes were shown in **Table 3**. The comparison between survivor and non-survivors was shown in **Table 4**. Multivariable cox regression analysis showed that neutropenia >7 days (P=0.030, HR: 4.068, 95%CI: 1.146~14.434), higher Pitt bacteremia score (P<0.001, HR:1.824, 95%CI: 1.322~2.517), higher Charlson comorbidity index (P=0.01, HR: 1.613, 95%CI: 1.124~2.315) and bacteremia due to MDR-PA (P=0.024, HR:3.086, 95%CI: 1.163~8.197) were independent risk factors of 30-day mortality (**Table 5**; **Figure 3**). We also compared the exact diagnoses and treatments of primary disease between survivors and non-survivors, but no significant differences were found (**Supplement File Table 1**).

**Table 4:** Characteristics of Patients with CRPA Bacteremia in Survivor and Non-survivor Groups.

Characteristic	All patients (n=100)	Survivor (n=79)	Non-survivor (n=21)	P value, univariable analysis
Demographic characteristic				
Age, years, median (IQR, range)	38.5(IOR: 26.3~51.8, range 3.0~69.0)	36(IOR: 26.0~51.0, range 60.0~66.0)	43(IOR: 30.5~54.5, range 3.0~69.0	0.284
Age ≥ 18, n (%)	85(85.0)	66(83.5)	19(90.5)	0.655
Age ≥ 55, n (%)	18(18.0)	13(16.5)	5(23.8)	0.645
Male sex, n (%)	56(56.0)	41(51.9)	15(71.4)	0.109
Underlying diseases, n (%)				
AML	51(51.0)	45(57.0)	6(28.6)	*0.021*
ALL	28(28.0)	22(27.8)	6(28.6)	0.948
MPAL	2(2.0)	2(2.5)	0(0)	0.622
MDS	4(4.0)	3(3.8)	1(4.8)	0.617
Lymphoma	1(1.0)	1(1.3)	0(0)	0.790
LGLL	1(1.0)	0(0)	1(4.8)	0.210
MM	2(2.0)	1(1.3)	1(4.8)	0.378
SAA	8(8.0)	4(5.1)	4(19.0)	0.058
SAA-PNH	2(2.0)	1(1.3)	1(4.8)	0.378
PNH	1(1.0)	0	1(4.8)	0.210
The status of the underlying diseases				
CR, n (%)	67(67.0)	60(75.9)	7(33.3)	*0.000*
Allo-HSCT before CRPA bacteremia, n (%)	29(29.0)	24(30.4)	5(23.8)	0.555
Neutropenia ≤ 7 days after CRPA BSI, n (%)	45(45.0)	43(54.4)	2(9.5)	*0.000*
Duration of neutropenia before and after CRPA BSI, days, median (IQR, range)	17.0(IQR: 10.0~27.5, range: 0~115.0)	16.0(IQR: 10.0~26.0, range: 0~115.0)	20.0(IQR :9.5~43.0, range: 0~97.0)	0.196
Previous CRPA colonization, n (%)				
Yes	38(38.0)	25(31.6)	13(61.9)	0.011
Severity of bacteremia				
Shock, n (%)	8(8.0)	4(5.1)	4(19.0)	0.100
Pitt bacteremia score, median (IQR, range)	1.0(IQR: 0~1.8, range: 0~6.0)	1.0(IQR: 0~1.0, range: 0~4.0)	2.0(IQR: 1.0~3.0, range: 0~6.0)	*0.000*
Concurrent multisite infection, n (%)				
Pneumonia	48(48.0)	29(36.7)	19(90.5)	*0.000*
Urinary infection	6(6.0)	2(2.5)	4(19.0)	0.021
Central nervous system infection	3(3.0)	1(1.3)	2(9.5)	0.111
With CVC				
Yes, n (%)	91(91.0)	75(94.9)	16(76.2)	0.025
Comorbidities				
Hypoproteinemia, n (%)	54(54.0)	36(45.6)	18(85.7)	*0.001*
Diabetes mellitus, n (%)	13(13.0)	9(11.4)	4(19.0)	0.574
Charlson comorbidity index, median (IQR, range)	2.0(IQR: 2.0~3.0, range: 1.0~6.0)	2.0(IQR: 2.0~3.0, range: 1.0~6.0)	4.0(IQR: 2.5~5.0, range: 2.0~5.0)	*0.000*
Bacteremia due to drug-resistant strain, n (%)				
Bacteremia due to MDR-PA	45(45.0)	30(38.0)	15(71.4)	*0.006*
Bacteremia due to DTR-PA	7(7.0)	4(5.1)	3(14.3)	0.322
Treatment regimen, timing and length				
Empirical combination therapy, n (%)	18(18.0)	15(19.0)	3(14.3)	0.858
Definitive combination therapy, n (%)	59(59.0)	44(55.7)	15(71.4)	0.193
Receiving in-vitro active antibiotics within 48 h from onset of fever, n (%)	57(57.0)	51(64.6)	6(28.6)	*0.003*
Length of therapy, days, median (IQR, range)	10.0(IQR: 6.25~13.0, range: 3.0~33.0)	10.0(IQR: 8.0~13.0, range: 4.0~21.0)	8.0(IQR: 3.0~14.0, range: 3.0~33.0)	0.146
Total	100	79	21	

CRPA, carbapenem-resistant Pseudomonas aeruginosa; IQR, interquartile range; AML, acute myeloid leukemia; ALL, acute lymphocytic leukemia; MPAL, mixed phenotype acute leukemia; MDS, myelodysplastic syndrome; LGLL, large granular lymphocytic leukemia; MM, multiple myeloma; SAA, severe aplastic anemia; PNH, paroxysmal nocturnal hemoglobinuria; CR, complete response; allo-HSCT, allogenic-hematopoietic stem cell transplantation; CVC, central venous catheter; MDR-PA, multidrug-resistant Pseudomonas aeruginosa; DTR-PA, difficult to treat Pseudomonas aeruginosa.

**Table 5:** Risk Factors for Mortality in Patients with CRPA BSI and MDR-PA BSI (Cox regression analysis).

Variable	CRPA BSI		MDR-PA BSI	
	HR (95% CI)	P value	HR (95% CI)	P value
Neutropenia > 7 days from onset of BSI	4.068 (1.146~14.434)	0.030	3.417 (0.968~12.057)	0.56
Charlson comorbidity index	1.613 (1.124~2.315)	0.010	1.772 (1.270~2.473)	0.001
Pitt bacteremia score	1.824 (1.322~2.517)	<0.001	1.878 (1.402~2.516)	<0.001
Bacteremia due to MDR-PA	3.086 (1.163~8.197)	0.024		
CR		excluded		excluded
Concurrent pneumonia		excluded		excluded
Hypoproteinemia		excluded		excluded
Receiving in-vitro active antibiotics within 48 h from onset of fever		excluded		excluded

CRPA, carbapenem-resistant Pseudomonas aeruginosa; BSI, bloodstream infections; MDR-PA, multidrug-resistant Pseudomonas aeruginosa; CR, complete response.

After controlling for neutropenia >7 days, higher Pitt bacteremia score, higher Charlson comorbidity index and bacteremia due to MDR-PA, a subsequent multivariable cox regression analysis demonstrated definitive regimens containing CAZ-AVI were associated with lower mortality (P=0.016, HR: 0.150, 95%CI: 0.032~0.702) comparing to definitive therapy without CAZ-AVI (**Table 6**; **Figure 3**). Regimens containing aminoglycosides were correlated with increased mortality (P=0.029, HR: 3.499, 95%CI: 1.139~10.749) comparing to definitive therapy without aminoglycosides. However, regimens containing quinolones (P=0.213, HR: 1.784, 95%CI: 0.718~4.432), carbapenems (P=0.935, HR: 1.056, 95%CI: 0.285~3.912), traditional β-lactam/β-lactamase inhibitor (other than CAZ-AVI) (P=0.973, HR < 0.01), colistin (P=0.984, HR < 0.01) and cephalosporin (P=0.900, HR: 1.145, 95%CI: 0.138~9.519) were found to show similar mortality rates at 30 days comparing to those regimens not containing respective drugs. No statistically significant difference was observed in 30-day mortality between the group receiving monotherapy and group receiving combination therapy (P=0.212, HR: 1.948, 95%CI: 0.684~5.546). The impacts of empirical antimicrobial regimens on the mortality were also assessed and no significant difference was observed (**Table 7**).

**Table 6:** The Impacts of Definitive Antimicrobial Regimens on the Mortality of Patients with CRPA BSI (n=100).

Antimicrobial regimen	Mortality (n, %)	Univariable analysis	Multivariable cox regression analysis	
		P value	a*HR (95% CI)	P value
Monotherapy or combination				
Monotherapy	6/41 (14.6)			
Combination	15/59 (25.4)	0.193	1.948 (0.684~5.546)	0.212
CAZ-AVI				
Without	19/76 (25.0)			
With	2/24 (8.3)	0.081	0.150 (0.032~0.702)	0.016
Aminoglycosides				
Without	15/81 (18.5)			
With	6/19 (31.6)	0.345	3.499 (1.139~10.749)	0.029
Quinolones				
Without	10/63 (15.9)			
With	11/37 (29.7)	0.100	1.784 (0.718~4.432)	0.213
Carbapenems				
Without	17/73 (23.3)			
With	4/27 (14.8)	0.356	1.056 (0.285~3.912)	0.935
Traditional β-lactam/β-lactamase inhibitor				
Without	12/61 (19.7)			
With	9/39 (23.1)	0.683	<0.01	0.973
Colistin				
Without	19/94 (20.2)			
With	2/6 (33.3)	0.804	<0.01	0.984
Cephalosporin				
Without	16/93 (17.2)			
With	5/7 (71.4)	0.004	1.145 (0.138~9.519)	0.900

* : adjusting for neutropenia >7 days, higher Pitt bacteremia score, higher Charlson comorbidity index and bacteremia due to MDR-PA; CAZ-AVI, ceftazidime-avibactam.

CRPA, carbapenem-resistant Pseudomonas aeruginosa; BSI, bloodstream infections.

**Table 7:** The Impacts of Empirical Antimicrobial Regimens on the Mortality of Patients with CRPA BSI (n=100).

Antimicrobial regimen	Mortality (n, %)	Univariable analysis	Multivariable cox regression analysis	
		P value	a*HR (95% CI)	P value
Monotherapy or combination				
Monotherapy	18/83 (21.7)			
Combination	3/17 (17.6)	0.964	0.514 (0.137~1.936)	0.326
CAZ-AVI				
Without	21/96 (21.9)			
With	0/4 (0)	0.576	0	0.983
Aminoglycosides				
Without	17/73 (18.9)			
With	4/10 (40.0)	0.252	1.613 (0.433~6.011)	0.476
Quinolones				
Without	21/96 (21.9)			
With	0/4 (0)	0.576	0	0.982
Carbapenems				
Without	3/23 (11.5)			
With	18/74 (24.3)	0.169	1.056 (0.285~3.912)	0.935
Traditional β-lactam/β-lactamase inhibitor				
Without	21/86 (24.4)			
With	0/14 (0)	0.084	0	0.973
Colistin				
Without	21/99 (21.2)			
With	0/1 (0)	1.000	0	0.984
Cephalosporin				
Without	20/94 (21.3)			
With	1/6 (16.7)	1.000	1.145 (0.138~9.519)	0.900

* : adjusting for neutropenia >7 days, higher Pitt bacteremia score, higher Charlson comorbidity index and bacteremia due to MDR-PA; CAZ-AVI, ceftazidime-avibactam.

CRPA, carbapenem-resistant Pseudomonas aeruginosa; BSI, bloodstream infections.

#### 3.4.2 For MDR-PA

For 45 patients infected with MDR-PA bacteremia, 30-day mortality was 33.3% (15/45). 7-day and 30-day clinical cure rates were 53.5% (24/45) and 64.4% (29/45). Multivariable cox regression analysis revealed that higher Pitt bacteremia score (P<0.001, HR:1.878, 95%CI: 1.402~2.516), higher Charlson comorbidity index (P=0.01, HR: 1.772, 95%CI: 1.270~2.473) remained independent predictors of 30-day mortality (**Table 5**).

**Figure 4:**
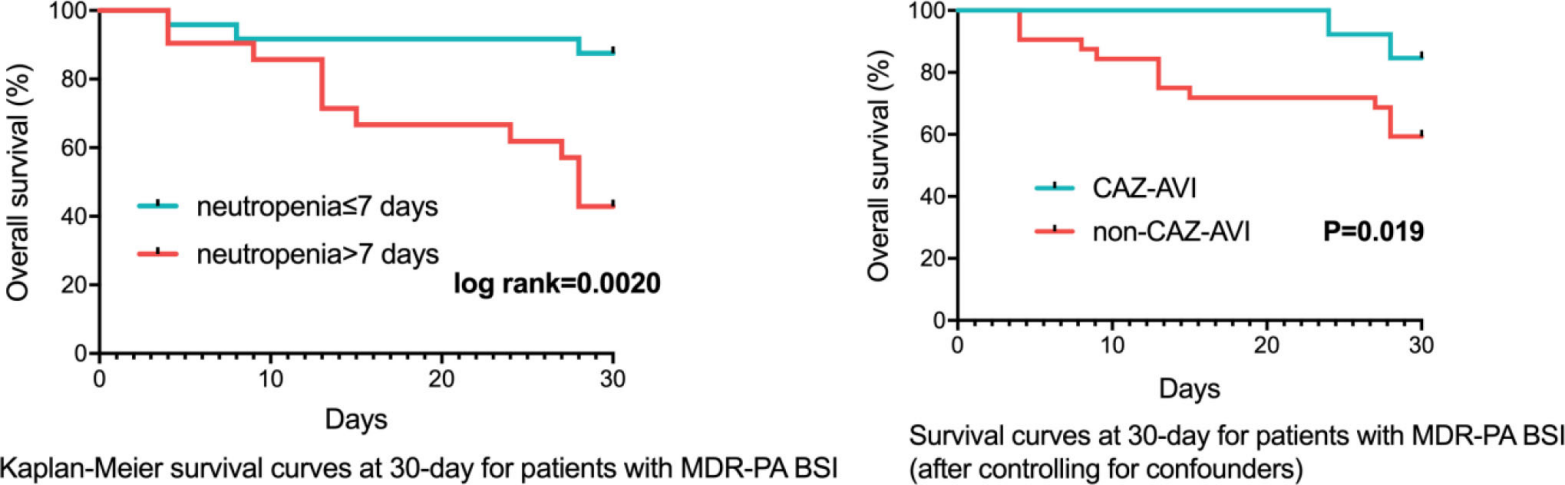
Survival Rates of MDR-PA BSI Patients (n=45). MDR-PA, multidrug-resistant Pseudomonas aeruginosa; BSI, bloodstream infections; CAZ-AVI, ceftazidime-avibactam.

Similar to CRPA, after controlling for confounders (neutropenia >7 days, higher Charlson comorbidity index and higher Pitt bacteremia score), a subsequent multivariable cox regression analysis demonstrated definitive regimens containing CAZ-AVI exhibited lower mortality (P=0.019, HR: 0.119, 95%CI: 0.020~0.709) comparing to those without CAZ-AVI (**Table 8**; **Figure 4**). Regimens containing aminoglycosides were correlated with higher mortality (P=0.006, HR: 6.457, 95%CI: 1.710~24.389) comparing to definitive therapy without aminoglycosides. No statistically significant difference in mortality rates was observed between patients with monotherapy and those with combination therapy (P=0.134, HR: 3.282, 95%CI: 0.694~15.520).

**Table 8:** The Impacts of Definitive Antimicrobial Regimens on the Mortality of Patients with MDR-PA BSI (n=45).

Antimicrobial regimen	Mortality (n, %)	Univariable analysis	Multivariable cox regression analysis	
		P value	a*HR (95% CI)	P value
Monotherapy or combination				
Monotherapy	3/10 (30.0)			
Combination	12/35 (34.3)	1.000	3.282 (0.694~15.520)	0.134
CAZ-AVI				
Without	13/32 (40.6)			
With	2/13 (15.4)	0.201	0.119 (0.020~0.709)	0.019
Aminoglycosides				
Without	9/32 (28.1)			
With	6/13 (46.2)	0.416	6.457 (1.710~24.389)	0.006
Quinolones				
Without	6/24 (25.0)			
With	9/21 (42.9)	0.205	2.531 (0.827~ 7.750)	0.104
Carbapenems				
Without	12/32 (37.5)			
With	3/13 (23.1)	0.561	1.147 (0.271~4.851)	0.852
Traditional β-lactam/β-lactamase inhibitor				
Without	10/29 (34.5)			
With	5/16 (31.3)	0.826	1.024 (0.335~3.126)	0.967
Colistin				
Without	11/41 (26.8)			
With	4/4 (100.0)	0.016	4.580 (0.823~25.481)	0.082
Cephalosporin				
Without	14/43 (32.6)			
With	1/2 (50.0)	1.000	1.245 (0.129~ 11.978)	0.850

*: adjusting for neutropenia >7 days, higher Pitt bacteremia score, higher Charlson comorbidity index and bacteremia due to MDR-PA.

MDR-PA, multidrug-resistant Pseudomonas aeruginosa; BSI, bloodstream infections.

#### 3.4.3 For DTR-PA

Notably, for 7 patients with DTR-PA bacteremia, 4 patients receiving CAZ-AVI were all still alive at 30-day, while the rest 3 patients treated with others (1 receiving colistin; 1 etimicin combination with ciprofloxacin; 1 meropenem, etimicin combination with ciprofloxacin) were deceased.

## 4 Discussion

This study reported 30-day mortality of CRPA BSI and MDR-PA BSI was 21.0% (21/100), 33.3% (15/45), respectively. Considering high mortality of infections due to antibiotic-resistant *Pseudomonas aeruginosa*, it is urgent to rapidly identify high-risk MDR infections, know about the risk factors of outcomes and choose effective antibiotics to improve survival. Both Viasus et al. and our recent study had developed a scoring system based on several clinical factors to recognize neutropenic patients who are at risk of developing BSI due to MDR-PA (Viasus et al., 2020; Zhao et al., 2022). In this study, we demonstrated neutropenia >7 days, higher Pitt bacteremia score, higher Charlson comorbidity index and bacteremia due to MDR-PA were independent risk factors of 30-day mortality for CRPA BSI patients. Moreover, this study also compared the efficacy of different antibiotic regimens. After controlling for confounders, CAZ-AVI-based regimens were significantly associated with increased survival rates, whereas aminoglycosides-based regimens were correlated with decreased survival rates.

Thirty-day mortality rates for CRPA BSI presented in this study (21.0%) were lower than those reported from prior studies, ranging from 30.0% to 68.3% (Joo et al., 2011; Peña et al., 2012; Dantas et al., 2014; Jeong et al., 2014; Buehrle et al., 2017; Papadimitriou-Olivgeris et al., 2017; Shi et al., 2019). The outcomes of CRPA BSI, similar to other infections, were affected by various factors including host factors, type of pathogen and medical interventions (Tong et al., 2012). For patients with hematological diseases, the prolonged and severe neutropenia probably exerted a significant impact on the efficacy of *Pseudomonas aeruginosa* clearance from the bloodstream. Bergas et al. (2022) revealed that profound neutropenia (<0.1×10^9^/L) was one of the independent risk factors contributing to 30-day mortality of *Pseudomonas aeruginosa* bacteremia in neutropenic hematologic patients (P=0.001, aOR: 5.49, 95%CI: 1.96~15.36). In our study, we explored the impact of neutropenia duration after BSI to clinical outcomes and found a significant increase in 30-day mortality among patients with neutropenia >7 days when compared to control group (34.5% (19/55) vs 4.4% (2/45), P<0.001). Multivariable analysis identified neutropenia >7 days as an independent risk factor of 30-day mortality for CRPA BSI patients (P=0.030, HR: 4.068, 95%CI: 1.146~14.434).

In the past, considering CRPA and MDR-PA isolates were always susceptible to aminoglycosides and polymyxins, regimens containing these antibiotics were regarded as feasible therapeutic options for individuals suspected of MDR-PA infections. Inmaculada et al (López Montesinos et al., 2022). conducted a comparative study to evaluate the efficacy of aminoglycosides or polymyxin monotherapy versus other antibiotics (aztreonam, carbapenems, cefepime, ceftazidime, ceftolozane-tazobactam, ceftazidime-avibactam) for the treatment of complicated urinary tract infections due to extensively drug-resistant *Pseudomonas aeruginosa*. They found colistin or aminoglycoside monotherapy did not exhibit a statistically significant association with an increased 30-day mortality (HR: 0.93, 95%CI: 0.17-5.08) or 90-day mortality (HR: 0.68, 95%CI: 0.20-2.31). Interestingly, in our study, when new antibiotic (CAZ-AVI) was included in the comparative group, regimens including aminoglycoside did not show good efficacy in treating BSI due to CRPA and MDR-PA.

As was mentioned before, novel antibiotic therapies including CAZ-AVI, ceftolozane-tazobactam support more options for CRPA and MDR-PA infections. Several real-world studies have exhibited favorable results of CAZ-AVI in treating MDR-PA infections (Jorgensen et al., 2019; Vena et al., 2020; Corbella et al., 2022). Chen et al. (2022) conducted a single-center study to compare the effectiveness of CAZ-AVI and polymyxin in treating CRPA infections. The study enrolled 136 patients, in which almost all patients had pneumonia infections (n=135, 99.3%) and 49 (36.0%) had bacteremia. The study indicated that CAZ-AVI group had a lower 14-day mortality rate (5.9% vs 27.1%, P=0.002) and 30-day mortality rate (13.7% vs 47.1%, P < 0.001) when comparing to polymyxin group. Multivariable analysis identified the use of CAZ-AVI as one of the independent predictors of 30-day survival. In our study, after adjusting for confounders, it was observed that comparing to traditional antibiotics, CAZ-AVI treatment regimen demonstrated a significantly reduced 30-day mortality for BSI due to CRPA (8.3% (2/24) vs 25.0% (19/76), P=0.016), as well as MDR-PA (15.4% (2/13) vs 40.6% (13/32), P=0.019). Therefore, our study supported more evidence that CAZ-AVI-based regimens were superior to traditional antibiotics regimens for patients with CRPA and MDR-PA BSI in the real world.

Infectious Diseases Society of America Guidance recommends using CAZ-AVI, ceftolozane-tazobactam, and imipenem-cilastatin-relebactam as preferred monotherapy options for treating DTR-PA infections occurring outside the urinary tract based on known *in vitro* activity (Tamma et al., 2021). But studies focusing on DTR-PA infection only limited to several case reports. In this study, for 7 patients with DTR-PA bacteremia, 4 patients receiving CAZ-AVI-based regimens were all alive at 30-day, while the rest 3 patients treated with others were deceased.

As is known to us, *Pseudomonas aeruginosa* exhibits complex mechanisms of resistance to the carbapenems. The common mechanisms of drug resistance include inactivation of the gene that codes for the porin OprD, overexpression of efflux systems and hyperproduction of the chromosomal cephalosporinase AmpC (Tenover et al., 2022). In addition, although less common, the production of carbapenemases is also an important mechanism of antibiotic resistance. which can alter the sensitivity of antimicrobial agents. For example, CAZ-AVI is active against class A, class D carbapenemases and AmpCs, but not against metallo beta lactamase (NDM, VIM, IMP) producers. The prevalence of carbapenemase genes in CRPA isolates differed depending on the region. Recently, an international prospective, multicenter cohort study collected 972 CRPA from bloodstream, respiratory, urine, or wound cultures of patients at 44 hospitals and reported that 211 (22%) were detected to harbor carbapenemase genes (Reyes et al., 2023). CRPA strains with the highest frequency of carbapenemase production were found in South and central America (88/127, 69%), followed by Australia and Singapore (32/56, 57%), China (54/171, 32%), Middle East (27/91, 30%) and the USA (10/527, 2%). As noted in our prior research (Zhao et al., 2022), none of the bloodstream-derived CRPA strains between October 2017 and April 2021 were found to produce carbapenemase. The result further demonstrated CAZ-AVI was effective against CRPA bloodstream in our hospital, which was consistent with the results of drug susceptibility testing. Detection of other resistance mechanisms of CRPA in our hospital, such as overexpression of efflux pumps, is still ongoing.

There exists an interesting phenomenon in this study. 18 patients received carbapenem as monotherapy (definitive therapy) despite the laboratory reporting CRPA. 17 of them survived for 30 days and one patient died. The antimicrobial therapy selections for survivors can be described as follows. When the fever occurred, blood cultures were taken and empirical treatment with carbapenem alone was used. If a decrease in body temperature and improvement in clinical symptoms were observed, empirical therapy was recognized as effective. Although susceptibility testing results were reported (approximately 2~3 days later), revealing resistance to carbapenem, we decided to maintain initial treatment in these patients considering its effectiveness. Additionally, one patient who died did not show improvement after being treated with carbapenem alone for three days. Before adjusting the treatment regimen to other effective antibiotics, the patient died of the worsening underlying disease and presence of multiple complications.

Regarding the potential effectiveness of carbapenems for the treatment of CRPA, we conducted an analysis of the underlying factors. Firstly, CRPA was defined as an isolate with MIC ≥ 4 ug/mL for imipenem or meropenem in accordance with the breakpoints of 2021 CLSI guidelines (CLSI, 2021). Among the 17 patients who survived CRPA infections, three received treatment with sensitive carbapenem. For example, when the cultured strain was resistant to imipenem but sensitive to meropenem, meropenem was selected as the treatment of choice. In addition, the administration of β-lactams can be optimized through higher doses and extended infusions, resulting in increases in the time that free drug concentrations remain above the MIC (fT>MIC). In animal infection models, carbapenems require at least 40% fT>MIC to achieve maximal bactericidal activity against *Pseudomonas aeruginosa* (Drusano, 2003). Many studies have reported that in pharmacokinetic simulation models, a 2g every 8h dose regimen of meropenem, with extended infusion or two-step-administration therapy (i.v. bolus plus prolonged infusion), had a high probability of attaining 40% fT>MIC against organisms (including *Pseudomonas aeruginosa*) with meropenem MICs of 4~8 ug/mL, even up to 16 ug/mL (Kuti et al., 2003; Bulik et al., 2010; Jaruratanasirikul et al., 2015; Song et al., 2019). Cojutti et al. (2020) conducted a prospective study to assess the effectiveness of real-time therapeutic drug monitoring-guided optimization of continuous-infusion meropenem in hematological patients with febrile neutropenia. Two patients had infections caused by CRPA. One patient achieved a pharmacodynamic target and was cured by meropenem monotherapy (meropenem MIC: 16 ug/mL); the other achieved a suboptimal pharmacodynamic targe and died (meropenem MIC: 32 ug/mL). These data indicated optimized carbapenem monotherapy displayed potential effective against CRPA with low-level resistance. Among 14 CRPA strains that received *in vitro* insensitive carbapenem treatment in this study, the median MIC value of meropenem for CRPA treated with meropenem was 4 ug/mL (MIC: 4 ug/mL, n=7; MIC: 8 ug/mL, n=1), while the median MIC value of imipenem for those treated with imipenem was 8 ug/mL (MIC: 8 ug/mL, n=4; MIC≥16 ug/mL, n=2), indicating a relatively low level of resistance. Of the 14 patients, four received high-dose imipenem (1.0g every 6h) or meropenem (2.0g every 8h) treatment. Further large-scale, comparative studies are required to investigate the efficacy of optimized carbapenem monotherapy for CRPA infections. Finally, previous researches have shown that host factors such as persistent neutropenia, septic shock, high Pitt bacteremia scores and severe underlying diseases are associated with outcomes among patients with CRPA infections (Buehrle et al., 2017; Bergas et al., 2022). Consistent with these findings, our study revealed that a rapid recovery from neutropenia, a lower Charlson comorbidity index, and lower Pitt bacteremia scores were all significantly associated with increased 30-day survival rates. Among the 17 surviving patients, 47.1% experienced neutropenia for less than 7 days after CRPA BSI. The median Charlson comorbidity index was 2 (IQR: 2~3; range: 2~6) and the median Pitt bacteremia score was 1 (IQR: 0~1; range: 0~2) for these patients (n=17). Conversely, the patient who died had neutropenia for more than 7 days, a Charlson comorbidity index of 3 and a Pitt bacteremia score of 6. In summary, in certain cases, optimizing carbapenem treatment may be effective against *Pseudomonas aeruginosa* exhibiting low-level resistance. Of particular note, in cases where empirical carbapenem treatment proves ineffective, prompt adjustment of therapeutic regimen is recommended.

The efficacy of combination therapy in improving survival of patients with *Pseudomonas aeruginosa* BSI remains a subject of debate. Prior retrospective cohorts and meta-analytic studies have reported no statistically significant difference in 30-day mortality between individuals receiving combination therapy and those receiving monotherapy (Hu et al., 2013; Peña et al., 2013; McCarthy et al., 2018). Kim et al. (2014) found combination therapy was beneficial in the group of *Pseudomonas aeruginosa* BSI patients with neutropenia. In our study, combination therapy did not provide significant benefits to hematological patients with CRPA BSI or MDR-PA BSI. However, it is important to note that in clinical setting, combination therapy is often preferred for patients with severe illness. Therefore, the effect of combination therapy in CRPA and MDR-PA BSI needs to be evaluated in randomized controlled trials.

There were several limitations in this study. Firstly, considering the retrospective nature and single-center design, it would include indication bias. Secondly, the number of CRPA samples, especially the numbers of patients receiving CAZ-AVI was relatively small, which limited the power of conclusion. Moreover, since CAZ-AVI was marketed in China in 2019, only a small part of strains was tested for drug sensitivity.

## 5 Conclusions

This study reported that 30-day mortality rates of CRPA BSI and MDR-PA BSI among patients with hematological diseases were high to 21.0% (21/100) and 33.3% (15/45). Neutropenia >7 days after BSI, higher Pitt bacteremia score, higher Charlson comorbidity index and bacteremia due to MDR-PA were independent risk factors of 30-day mortality for CRPA BSI patients. After controlling for confounders, CAZ-AVI-based regimens were effective alternatives for the treatment of bacteremia due to CRPA and MDR-PA. However, larger prospective cohort studies or randomized trials are needed to validate and expand upon the findings of this study.

## Data availability statement

The raw data supporting the conclusions of this article will be made available by the authors, without undue reservation.

## Ethics statement

The studies involving human participants were reviewed and approved by ethical committee of the Institute of Hematology and Blood Diseases Hospital, Chinese Academy of Medical Sciences. Written informed consent to participate in this study was provided by the participants’ legal guardian/next of kin.

## Author contributions

SF designed the study and revised the manuscript. SZ wrote the manuscript. YZ analyzed the data. SZ, YZ, ZC, TZ and JRW collected the data. EJ, FZ, YM, XZ, MH, ZX, JXW and SF provided patients to study. All authors contributed to the article and approved the submitted version.

## Supplementary material

Supplementary Table 1 Various Disease Types and Disease-specific Treatments Among the 100 patient Cohort.
