# Supplementary material for: Assessment of mortality-related risk factors and effective antimicrobial regimens for treatment of bloodstream infections caused by carbapenem-resistant Pseudomonas aeruginosa in patients with hematological diseases
Source: Front Cell Infect Microbiol. 2023 Jun 21;13:1156651. doi: 10.3389/fcimb.2023.1156651 (PMC10320591; doi:10.3389/fcimb.2023.1156651)
Supplement: Supplementary Table 1 — Various Disease Types and Disease-specific Treatments Among the 100 patient Cohort. [file Table_1.docx]

**Supplement File**

**Table 1. Various Disease Types and Disease-specific Treatments Among the 100 patient Cohort.**

| **Diagnosis** | **Survivor** | **Non-survivor** | **P value, univariable analysis** |
| --- | --- | --- | --- |
| **AML (n=51)** | **n=45** | **n=6** |  |
| Cytogenetics (**ELN 2022 risk stratification)** |  |  | 0.861 |
| Favorable | 11(24.4) | 1(16.7) |  |
| Intermediate | 21(46.7) | 4(66.7) |  |
| Adverse | 13(28.9) | 1(16.7) |  |
| **Therapy phase at BSI** |  |  | 0.061 |
| Induction therapy | 14(31.1) | 5(83.3) |  |
| Chemotherapy-based consolidation therapy or maintenance therapy | 19(42.2) | 0 |  |
| Allo-HSCT | 11(24.4) | 1(16.7) |  |
| Auto-HSCT | 1(2.2) | 0 |  |
| **ALL (n=28)** | **n=22** | **n=6** |  |
| Diagnosis |  |  | 0.493 |
| Ph positive B-ALL | 5(22.7) | 1(16.7) |  |
| Ph negative B-ALL | 12(54.5) | 2(33.3) |  |
| T-ALL | 5(22.7) | 3(50.0) |  |
| **Therapy phase at BSI** |  |  | 0.339 |
| Induction therapy | 6(27.3) | 4(66.7) |  |
| Chemotherapy-based consolidation therapy or maintenance therapy | 9(40.9) | 2(33.3) |  |
| Allo-HSCT | 6(27.3) | 0 |  |
| Auto-HSCT | 1(4.5) | 0 |  |
| Prior TKI exposure |  |  | 1.000 |
| Yes | 5(22.7) | 1(16.7) |  |
| No | 17(77.3) | 5(83.3) |  |
| **MPAL (n=2)** | **n=2** | **n=0** |  |
| Diagnosis |  |  | -- |
| Ph positive | 1(50.0) | 0 |  |
| Ph negative | 1(50.0) | 0 |  |
| MDS (n=4) | **n=3** | **n=1** |  |
| Diagnosis（WHO 2022） |  |  | 1.000 |
| MDS-LB | 1(33.3) | 1(100.0) |  |
| MDS-IB | 2(66.7) | 0 |  |
| Risk prediction (IPSS-M 2023) |  |  | 0.250 |
| low | 0 | 1(100.0) |  |
| high | 3(100.0) | 0 |  |
| **Therapy regimen at BSI** |  |  | -- |
| Allo-HSCT | 3(100.0) | 1(100.0) |  |
| **Lymphoma (n=1)** | **n=1** | **n=0** |  |
| DLBCL | 1 | 0 |  |
| **LGLL (n=1)** | **n=0** | **n=1** |  |
| **MM (n=2)** | **n=1** | **n=1** |  |
| **SAA (n=8)** | **n=4** | **n=4** |  |
| Diagnosis |  |  | 1.000 |
| VSAA | 2(50.0) | 3(75.0) |  |
| Non-VSAA | 2(50.0) | 1(25.0) |  |
| **Therapy regimen at BSI** |  |  | 1.000 |
| Allo-HSCT | 1(25.0) | 0 |  |
| ATG+CsA+others | 1(25.0) | 2(50.0) |  |
| CsA+others | 2(50.0) | 1(25.0) |  |
| others | 0 | 1(25.0) |  |
| **SAA-PNH (n=2)** | **n=1** | **n=1** |  |
| **PNH (n=1)** | **n=0** | **n=1** |  |

Abbreviations: AML, acute myeloid leukemia; **ELN, t**he European Leukemia Net; **BSI,** bloodstream infections; Allo-HSCT, allogenic hematopoietic stem cell transplantation; Auto-HSCT, autologous hematopoietic stem cell transplantation; ALL, acute lymphocytic leukemia; Ph, Philadelphia chromosome; B-ALL, B-cell acute lymphoblastic leukemia; T-ALL, T-cell acute lymphoblastic leukemia; TKI, tyrosine kinase inhibitor; MPAL, mixed phenotype acute leukemia; MDS, myelodysplastic syndrome; MDS-LB, ﻿myelodysplastic syndrome with low blasts; MDS-IB, ﻿myelodysplastic syndrome with increased blasts; IPSS-M, Molecular International Prognostic Scoring System; DLBCL, diffuse large B-cell lymphoma; LGLL, large granular lymphocytic leukemia; MM, multiple myeloma; SAA, severe aplastic anemia; VSAA, very severe aplastic anemia; ATG, antithymocyte globulin; CsA, cyclosporine; PNH, paroxysmal nocturnal hemoglobinuria;
